# Supplementary material for: Identification of MicroRNA Expression Profiles Related to the Aggressiveness of Salivary Gland Adenoid Cystic Carcinomas
Source: Genes (Basel). 2023 Jun 2;14(6):1220. doi: 10.3390/genes14061220 (PMC10298384; doi:10.3390/genes14061220)
Supplement: Supplementary file 1 [file genes-14-01220-s001.zip › genes-2327655-supplementary.pdf]

Supplementary Materials: Identification of MicroRNA Expression Profiles Related to the Aggressiveness of Salivary Gland Adenoid Cystic Carcinomas

Maicon Fernando Zanon [1], Cristovam Scapulatempo-Neto [1], Ricardo Ribeiro Gama [2], Márcia Maria Chiquitelli Marques [1], Rui Manuel Reis [1,3,4], and Adriane Feijó Evangelista [1,5*]

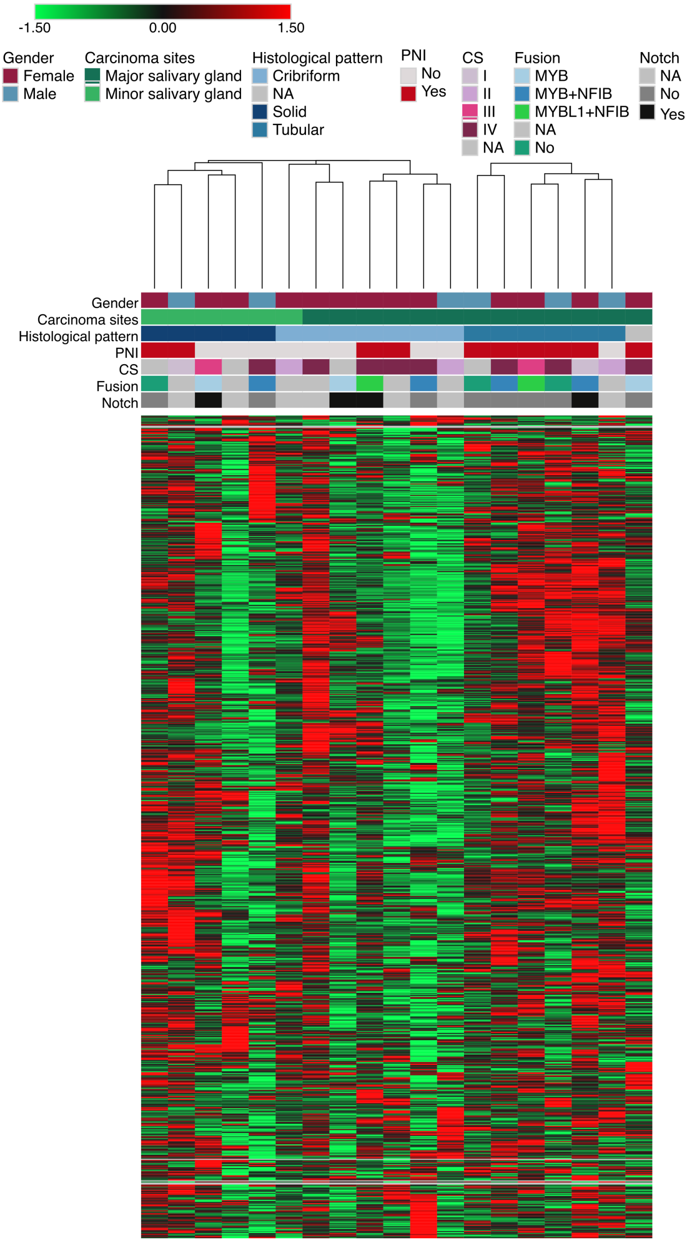

Figure S1. Unsupervised clustering of microRNAs of patients with ACC. The red and green colors indicate up-regulation and down-regulation of miRNAs. The positivity for *NOTCH1* and fusion genes were determined according to the average plus or minus two standard deviations of expression values of all the samples.
